# Measurement-Based Care in a Remote Intensive Outpatient Program: Pilot Implementation Initiative

**DOI:** 10.2196/58994

**Published:** 2024-10-23

**Authors:** Komal Kumar, Amber W Childs, Jonathan Kohlmeier, Elizabeth Kroll, Izabella Zant, Stephanie Stolzenbach, Caroline Fenkel

**Affiliations:** 1 Charlie Health, Inc Bozeman, MT United States; 2 Department of Psychiatry Yale School of Medicine New Haven, CT United States

**Keywords:** measurement-based care, MBC, remote, intensive outpatient program, IOP, mental health, implementation

## Abstract

**Background:**

The ongoing mental health crisis, especially among youth, has led to a greater demand for intensive treatment at the intermediate level, such as intensive outpatient programs (IOPs). Defining best practices in remote IOPs more broadly is critical to understanding the impact of these offerings for individuals with high-acuity mental health service needs in the outpatient setting. Measurement-based care (MBC), or the routine and systematic collection of patient-reported data throughout the course of care to make meaningful changes to treatment, is one such practice that has been shown to improve patient outcomes in mental health treatment. Despite the literature linking MBC to beneficial clinical outcomes, the adoption of MBC in clinical practice has been slow and inconsistent, and more research is needed around MBC in youth-serving settings.

**Objective:**

The aim of this paper is to help bridge these gaps, illustrating the implementation of MBC within an organization that provides remote-first, youth-oriented IOP for individuals with high-acuity psychiatric needs.

**Methods:**

A series of 2 quality improvement pilot studies were conducted with select clinicians and their clients at Charlie Health, a remote IOP program that treats high-acuity teenagers and young adults who present with a range of mental health disorders. Both studies were carefully designed, including thorough preparation and planning, clinician training, feedback collection, and data analysis. Using process evaluation data, MBC deployment was repeatedly refined to enhance the clinical workflow and clinician experience.

**Results:**

The survey completion rate was 80.08% (3216/4016) and 86.01% (4218/4904) for study 1 and study 2, respectively. Quantitative clinician feedback showed marked improvement from study 1 to study 2. Rates of successful treatment completion were 22% and 29% higher for MBC pilot clients in study 1 and study 2, respectively. Depression, anxiety, and psychological well-being symptom reduction were statistically significantly greater for MBC pilot clients (*P*<.05).

**Conclusions:**

Our findings support the feasibility and clinician acceptability of a rigorous MBC process in a real-world, youth-serving, remote-first, intermediate care setting. High survey completion data across both studies and improved clinician feedback over time suggest strong clinician buy-in. Client outcomes data suggest MBC is positively correlated with increased treatment completion and symptom reduction. This paper provides practical guidance for MBC implementation in IOPs and can extend to other mental health care settings.

## Introduction

### Background

As the mental health crisis persists and escalates, the Centers for Disease Control and Prevention report that the second leading cause of death for youth and young adults aged 10 to 24 years is suicide, with suicide rates for this age group having increased by 52.2% between 2000 and 2021 [1]. Moreover, recent years marked by the COVID-19 pandemic have witnessed a significant surge in the prevalence of mental health disorders across all age groups, with the pandemic having led to an overall 28% increase in cases of major depressive disorder and a 26% increase in cases of anxiety disorders worldwide in 2020 [2]. During the COVID-19 pandemic, more than 33% of high school students experienced poor mental health and nearly half of them reported feeling persistently sad or hopeless [3]. In particular, while the need for mental health services is increasing, there is a significant gap in service use, especially for vulnerable youth in the United States [3,4].

Within the context of exacerbated mental health issues, there has been a corresponding increase in the need for intensive treatment beyond what is provided in standard outpatient care, but not to the level of residential or inpatient care. An intensive outpatient program (IOP) is one such intermediate level of care. Although models may vary, generally, IOPs include some combination of individual and group-based psychotherapy, medication management, and care coordination services [5,6]. While IOPs were traditionally in person before 2020, the pandemic ushered in a new age of remote care options, extending beyond just one-to-one outpatient offerings to include group-based offerings, such as IOPs. Given that over half of the US population (53%) lives in mental health provider shortage areas where access to mental health providers is sparse [7], one of the primary benefits of remote care is accessibility. Beyond the practical advantages of remote care, research has shown that remote care is comparable to in-person care in terms of both clinical outcomes and patient satisfaction [8,9].

While the literature is building [10-12], there is an ongoing need to understand best practices within the IOP level of care more broadly and within remote IOPs specifically [6]. One such practice is that of measurement-based care (MBC), an important evidence-based practice (EBP) requiring ongoing examination within youth-serving IOP settings. MBC is the process of routinely and systematically gathering and using patient-reported data to track patient progress and customize treatment [13,14]. The clinical process of MBC can be described as “collect, share, and act” [14] in which (1) “collect” refers to the routine collection of patient-reported outcomes measures, throughout treatment; (2) “share” describes engaging the patient in collaborative discussion regarding their scores and symptom trends; and, finally, (3) “act” involves leveraging data to guide treatment planning and make meaningful changes to care.

Meta-analytic and summary reviews of the literature have linked MBC to improved clinical outcomes over usual care [15], an increased ability to detect when patient progress is not on track [16], and reduced rates of treatment dropout [17]. MBC has also been associated with an improved therapeutic alliance, prompting transparent communication, and has been proposed to have the potential to address health disparities by encouraging shared decision-making between providers and patients from racially disadvantaged groups [18,19].

However, despite the numerous benefits of MBC, as well as organizational accountability mandates [20], such as that issued by the Joint Commission requiring behavioral health care organizations (BHCOs) to use MBC, the actual practice of MBC continues to be slow and inconsistent [21,22]. Known challenges to MBC implementation at the patient, provider, and system levels have been comprehensively documented within the building MBC literature [23,24]. Furthermore, while the literature examining applications of MBC within youth-serving settings is building, especially in traditional outpatient mental health care contexts [25-27], ongoing work is needed, particularly within the context of intermediate levels of care (eg, IOPs) in the remote-first space. Namely, practical implementation guidance and support to effectively incorporate MBC into care delivery is needed. Moreover, examination of MBC implementation within the context of remote care models exclusively is needed, particularly as the landscape of mental health care increasingly includes remote-first treatment opportunities [28,29].

Despite the known challenges to MBC implementation, there are a number of strategies that have shown promise in other settings, such as (1) assessing barriers and facilitators before the implementation; (2) identifying key personnel and preparing a usable implementation plan [30]; (3) involving providers in decisions about implementation, workflow, and process clarity; and (4) developing an implementation support plan involving clinical leadership and local champions [31]. This paper, a quality improvement (QI) initiative, builds on some of these strategies and adds to the literature by illustrating the implementation and clinical outcomes of MBC within an organization that provides remote-first, youth-oriented IOP services for individuals with high-acuity psychiatric service needs.

### Current Initiative

The current initiative was conducted within Charlie Health, a national remote IOP currently serving high-acuity teenagers and young adults aged 11 to 34 years across 33 states. Before the current implementation, there was a data-driven protocol in place at Charlie Health where a self-report measure (the World Health Organization-Five Well-Being Index [WHO-5] [32,33], a 5-item scale measuring subjective well-being over the last 2 weeks) was being collected within the context of individual psychotherapy sessions. However, there were a number of prevailing challenges, including (1) a lack of consistency in the process and use of the measure across clinicians, (2) clinicians citing the WHO-5 as being limited in its ability to guide weekly conversations and treatment planning with clients, and (3) clinicians wanting more training around incorporating data into their therapeutic approaches. Thus, clinical leadership was invested in launching a more unified and robust MBC process under the “Collect, Share, and Act” [14] model, leading to the current initiative consisting of 2 pilot studies.

At Charlie Health, clients present with a range of mental health disorders, with the 2 most common primary diagnoses being major depressive disorder and generalized anxiety disorder. The core program includes a holistic and interdisciplinary approach to care, consisting of the following: 9 hours of evidence-based psychotherapy groups, split into three 3-hour sessions per week; 1 hour of individual therapy weekly; 1 hour of optional family therapy weekly; medication management and consultation as needed; and case management and referral services. Cohort placements for group therapy sessions are based on a variety of factors, including condition, appropriate therapeutic modalities, age, and lived experience. All clients receive a primary clinician, the same clinician they see in individual and family therapy sessions, as their main point of contact throughout the duration of their treatment. All treatment sessions are conducted on Zoom (Zoom Video Communications, Inc), a videoconferencing platform familiar to most clients, with enhanced encryption features to ensure privacy and security. The average length of stay in the program is 10 to 12 weeks.

Structurally, clinicians and their clients are organized into regional clinical cohorts, each comprising approximately 20 to 30 clinicians, overseen by a clinical director and 1 to 2 clinical supervisors. These clinical cohorts convene weekly for team meetings, enabling the exchange of updates, case consultations, and the opportunity to address queries collectively. The organizational structure of our clinical teams proves advantageous for implementing pilot initiatives, as specific cohorts can be selected for targeted projects. This approach offers multiple benefits: clinicians can be trained and communicated with selectively, A/B testing can easily be conducted, and clinical outcomes can be compared between clinical cohorts.

In the following implementation, we used the updated Consolidated Framework for Implementation Research (CFIR) [34] as a theoretical underpinning to structure the initiative. While a detailed review of the model is beyond the scope of this paper, the CFIR provides a thorough structural and theoretical framework aimed at facilitating the integration of EBPs into patient care [35]. CFIR consists of five main domains: (1) *innovation* or the characteristics of the thing being implemented; (2) *outer setting* or the external factors impacting the implementation; (3) *inner setting* or the setting in which the innovation is actually being implemented; (4) *individuals* or the roles and characteristics of the individuals involved; and (5) *implementation process* or the activities and strategies used to implement the innovation. While we did not use the full array of constructs outlined by the CFIR, in keeping with the flexible nature of the framework, we noted aspects of CFIR that were most relevant and applicable to the current setting throughout the paper.

## Methods

### Study 1

#### Ethical Considerations

This study retrospectively evaluated data from patients whose treatment had ended primarily to inform the improvement of ongoing programing. Patients give authorization for their surveys and recorded clinical data to be used for ongoing program evaluation when they agree to treatment; they are provided this information again each time they are provided a survey, and have the option to skip the full survey and/or any individual questions they choose.

Given that the client data used for the analyses in this paper were observational and pulled from our existing electronic health record system and that the primary purpose was QI rather than research, the initiative was reviewed and approved by the NorthStar Institutional Review Board as exempt from the common rule definition of research (NB400174). The primary client consent covers secondary analysis without additional consent. Patients received no compensation for their participation in the study.

#### Key Personnel

Three key decisions were made at the outset concerning the individuals who would be involved in the initiative (CFIR *implementation leads* construct). First, an external subject matter expert (SME), the second author, was engaged to provide consultation and the necessary clinician training. The second decision was to assemble a task group with relevant areas of expertise across the organization, including 15 leaders from the following departments: (1) clinical; (2) research and clinical outcomes; (3) care delivery; (4) compliance; (5) utilization review; (6) engineering, product, and design; and (7) growth strategy. The third decision involved appointing a project manager with a background in clinical research to lead the task group and oversee the implementation from start to finish.

#### Measurement Selection and Process Development

In determining the measurement set, we selected standardized measures appropriate for a younger population, targeting the most commonly experienced symptoms among our clients. We also prioritized measures that were brief, free of cost, easily understood and explained, and available in the public domain [31]. In collaboration with the chief clinical officer and cofounder, 2 clinical directors, and the research and clinical outcomes team, we chose the following instruments: the (1) WHO-5 (psychological well-being) [32]; (2) PROMIS (Patient-Reported Outcomes Measurement Information System) Pediatric Peer Relationships Scale [36]; (3) PROMIS Pediatric Family Relationships Scale [37]; (4) PROMIS Pediatric Depressive Symptoms Short Form [38]; and (5) PROMIS Pediatric Anxiety Symptoms Short Form [38]. To cater to the diverse age groups within our client base, we opted for the pediatric versions of the 4 PROMIS scales for adolescents (<18 years) and the adult versions for young adult clients (≥18 years).

Given that the total number of items across the 5 measures was 33, resulting in a lengthy and potentially burdensome weekly survey, we split the measures into 2 different surveys administered on alternating weeks. The WHO-5 and Peer and Family Relationships Scales (survey 1) were administered on odd weeks, while the depression and anxiety measures (survey 2) were administered on even weeks.

**Figure 1:**
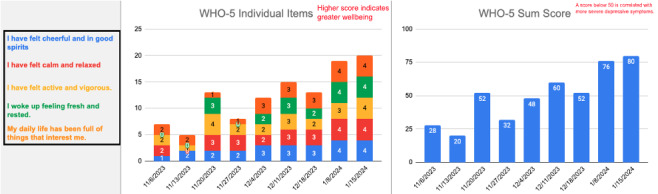
Sample data display graphs for the World Health Organization- Five Well-Being Index (WHO-5) scale created in preparation for the launch of study 1.

Next, in collaboration with our engineering, product, and clinical teams, technical and workflow components were established. Surveys were administered using Qualtrics (Qualtrics, LLC) alongside an internal solution for real-time display of responses within sessions. For each measure, 2 graphs were generated—a sum score graph and an individual items graph—to showcase historical data, encompassing all previous weeks, alongside the most recent set of scores. A set of sample graphs for the WHO-5 scale is included in Figure 1. Graphs were designed to be intuitive and easy to follow, so clinicians and clients could view and discuss the results on their screen during the session and collaboratively make decisions based on the data.

#### Cohort Characteristics

In pilot study 1, we selected 2 clinical cohorts totaling 27 clinicians, each working with around 20 to 25 clients. This resulted in approximately 600 clients in the study at any given time. As mentioned, clinical cohorts were divided by region; the 2 clinical cohorts chosen for this study were selected to allow for the broadest geographical representation of clients.

The inclusion of the clinical directors from both clinical cohorts in the task group proved pivotal during the planning phase, as they provided valuable insights into their respective team cultures and practices (CFIR *culture* construct). To further understand clinician attitudes and inform the training approach, we conducted the 15-item Evidence-Based Practice Attitudes Scale (EBPAS-15) [39] before developing the trainings. This 15-item tool assesses clinician attitudes regarding the implementation of EBPs in mental health practice. Along with a total score, the tool yields four domain scores: (1) *appeal* or the extent to which a clinician may adopt an EBP because they find it appealing or if trusted peers and colleagues are using the intervention; (2) *requirements* or the likelihood of adopting the EBP given the requirements to do so; (3) *openness* or the extent to which the clinician is open to new clinical interventions; and (4) *divergence* or the clinician’s perception of the degree to which the novel EBP differs from their current clinical practice. Items are rated on a Likert scale from 0 (not at all) to 4 (very great extent).

EBPAS-15 scores for this group of clinicians (Table 1) were 2.8 (moderate extent) in the *appeal* domain; 2.9 (moderate extent) in the *requirements* domain; 3.0 (great extent) in the *openness* domain; and 0.7 (not at all) in the *divergence* domain. Average domain scores revealed similar attitudes toward the implementation of EBPs between this cohort of clinicians and previously published samples of clinicians [40,41]. Internal consistency (Cronbach α) for the EBPAS-15 global scale was good (α=0.81) and subscales ranged from acceptable to excellent (required=0.98; appeal=0.88; openness=0.85; and divergence=0.73). These values indicate similar or slightly stronger internal consistency than in previously reported scores, such as globally α=0.77, 0.79 [40], and 0.82 [41].

The assessment revealed positive attitudes toward implementing EBPs, which lent itself well to the implementation of MBC with this group of clinicians. Given that the openness domain score averaged 3.0, indicating a high level of receptiveness to new clinical interventions, we opted to present the trainings as opportunities to acquire valuable new clinical skills, rather than emphasizing their mandatory nature.

**Table 1:** 15-Item Evidence-Based Practice Attitudes Scale (EBPAS-15) scores from clinicians in both study 1 (n=27) and study 2 (n=25), conducted before the start of each cohort’s trainings.

EBPAS-15 domain	Score range	Study 1 domain score, mean (SD)	Study 2 domain score, mean (SD)
Appeal	0-4^a^	2.8 (1.1)^b^	2.4 (1.1)^b^
Requirements	0-4^a^	2.9 (0.9)^b^	3.2 (0.7)^c^
Openness	0-4^a^	3.0 (0.8)^c^	3.1 (0.7)^c^
Divergence	0-4^d^	0.7 (0.9)^e^	0.7 (0.9)^e^

^a^Higher is better.

^b^Moderate extent.

^c^Great extent.

^d^Lower is better.

^e^Not at all.

#### Training and Launch

Before the start of MBC training, the clinical directors emphasized the importance of socializing MBC to the team, aiming to foster enthusiasm and create a positive mindset for clinicians going into the training series. To do this, our cofounder and chief clinical officer attended weekly team meetings 2 weeks before the trainings began, providing a preview and building excitement for the upcoming training series.

The training spanned 4 hours, divided into two 2-hour sessions, and was facilitated live by the external SME. Mandatory attendance for clinicians was closely monitored by the clinician experience team. Both segments of the training, part 1 and part 2, were recorded for future reference and for those unable to attend either portion of the training. Individuals who did not attend the live training were assigned to watch the recording before the official launch date. Part 1 of the training covered the first 2 components of the MBC process, namely “collect” and “share.” Part 2 covered the last component, “act,” along with the technical workflow. Both sessions included lecture segments, interactive group polls and quizzes, practice and role-plays in smaller groups, and opportunities for questions and answers.

Clinicians were trained at the end of the MBC core trainings to execute four main steps: (1) at the start of each session, send the client their designated MBC survey link (survey 1 or survey 2, depending on the treatment week) for private completion on their own screens (“collect”); (2) upon the client’s survey submission, access the data display interface and load the client’s graphs; (3) share the graphs with the client during the session, using the screenshare feature on Zoom, and discuss the various components (“share”); and (4) determine next steps in collaboration with the client and document the conversation, along with any actions or adjustments to the treatment plan influenced by the MBC scores in the client chart after the session (“act”). A key point of emphasis throughout the training was the importance of elevating the client’s voice throughout the MBC process, particularly in the “share” and “act” steps. This involved gaining a deep understanding of the client’s perspectives on their own data, ensuring their insights and experiences were integral to decision-making.

Once clinicians were trained and ready to start using MBC in their individual sessions, pilot study 1 was officially launched on February 13, 2023.

#### Feedback Collection

Given that a major goal of this study was collecting and implementing feedback to refine the process before its widespread implementation across all care teams, consistent feedback collection played a significant role in the months following the launch (CFIR *reflecting and evaluating* construct). This regular and reciprocal feedback was a key driver of the evaluation and reflection process, as well as the adaptation process (CFIR *innovation adaptability* construct).

Feedback was obtained in 2 main ways. First, the clinical directors and supervisors of each clinical cohort, who met with each clinician on their team on a weekly basis, would check-in with each clinician regarding their experience with MBC, including any successes, challenges, and opportunities for improvement. These clinical leaders would then convey this feedback in weekly task group meetings, allowing the team to discuss possible changes and iterations. Second, and perhaps most impactfully, the project manager met with the entire pilot cohort on a monthly basis to facilitate open dialogue regarding the pilot. In each of these feedback sessions, quantitative and qualitative feedback was collected through a short survey at the start of the session and key points from the group conversations were noted for further examination. Since each clinical cohort already held weekly team meetings, no additional sessions needed scheduling, making it convenient for all pilot clinicians to participate.

#### Data Analysis

Several data sources were analyzed to determine implementation success and outcomes.

##### Survey Completion Rate

One important metric for evaluating clinician fidelity to the “collect” portion of the model was the survey completion rate, computed by dividing the number of MBC assessments completed by pilot clinicians’ clients by the total number of sessions the clinician conducted in a week.

##### Monthly Feedback Survey

Another helpful indicator of pilot success was the monthly feedback collected live in cohort meetings. In each feedback session, a survey was administered, consisting of several quantitative items and 2 qualitative questions. For the quantitative questions, we used an internally developed 7-item scale to understand clinician attitudes toward the MBC implementation, where items such as “MBC adds value to my clinical practice” and “I like using MBC” are scored on a 5-point scale ranging from 1 (“completely disagree”) to 5 (“completely agree”). Average scores were calculated for each individual item. Regarding the qualitative questions, one inquired about challenges and the other about successes, and qualitative coding was performed to extract major themes. Given the small sample sizes of clinicians, instead of using formal tools or software, we used a straightforward approach where we manually reviewed the data and quantified the frequency of mentions for each major theme.

##### Client Outcomes

###### Overview

By comparing outcomes of clients who used the pilot version of MBC during their treatment against clients who received treatment as usual (TAU), we gained valuable insights into how MBC corresponds to treatment outcomes. To mitigate the impact of variations in clinical teams, such as differences in regions served and team culture or leadership, on client outcomes, our analysis zeroed in on comparing clients within the same clinical teams. At the time of the analysis, the 2 initial clinical cohorts selected for the study had expanded significantly. The original 27 pilot clinicians now constituted only half of their respective clinical cohorts, as new clinicians had been onboarded and some clinicians had been transferred from other cohorts. This meant that within the 2 clinical teams, half of the clinicians had undergone training and were actively using the MBC process with all their clients (MBC group), while the remaining half had not received this training, so their clients were not receiving the MBC program (TAU group).

One key metric, pulled from client charts, is discharge type. For this analysis, we recoded discharge type into a binary variable, with “routine discharge” referring to clients discharged due to successful treatment completion and “nonroutine discharge” referring to clients discharged for other reasons such as disengagement or moving to a higher or lower level of care. We then compared the rate of routine discharge between groups.

To assess client-reported outcomes, we used 3 scales from a typical assessment that all clients complete at intake and discharge. For each scale, we calculated average scores at intake and discharge and used 2-tailed independent samples *t* testing to compare symptom reduction scores between the MBC and TAU groups. All analyses were performed using SPSS (version 29; IBM Corp).

###### Depression

To measure depressive symptoms, we used the 9-item Patient Health Questionnaire Modified for Adolescents (PHQ-9) [42]. Ratings range from 0 (“not at all”) to 3 (“nearly every day”); item scores were added to produce a sum score, ranging from 0 to 27. Sum score cutoffs of 5, 10, 15, and 20 represented mild, moderate, moderately severe, and severe depression, respectively.

###### Anxiety

To measure anxiety symptoms, we used the 7-item Generalized Anxiety Disorder [GAD-7] [43], in which item ratings range from 0 (“not at all”) to 3 (“nearly every day”). Sum scores range from 0 to 21, and score cutoffs of 5, 10, and 15 represent mild, moderate, and severe anxiety, respectively.

###### Psychological Well-Being

To measure psychological well-being, we used the WHO-5 [32], in which item ratings range from 0 (“at no time”) to 5 (“all of the time”). Raw sum scores range from 0 to 25, which are multiplied by 4 to obtain a final percentage score. A percentage score of 0 represents the worst possible quality of life, whereas a score of 100 represents the best possible quality of life.

### Study 2

#### Overview

To apply the learnings from study 1 before initiating widespread implementation across all care teams, a second study was initiated with a new clinical cohort, which officially launched on September 25, 2023. Most of the methods from study 1 were replicated, with key modifications outlined in Table 2 (key learnings 1-4). Notably, clinicians in study 2 used the updated measurement set and administered the same survey on a weekly basis.

**Table 2:** Summary of key learnings and changes implemented from study 1 and study 2, collected from multiple avenues of clinician feedback.

Study and learning		Theme	Description	Change implemented	Recommendations
**Study 1**					
	1	Measurement set optimization	The peer and family relationship scales were static in nature and limited in reflecting weekly improvements. Clinicians also deemed these scales irrelevant to many clients and highlighted the need for a measure evaluating activities of daily living [44] instead.	The peer and family scales were replaced with an internally devised 6-item functional wellbeing scale, measuring: attendance at school and work, sleep, hygiene, eating, and medication adherence.	Measurement set must be sensitive to change, especially in intensive outpatient programs where sessions are frequent.
	2	Survey cadence simplification	The alternating survey administration was cumbersome and ineffective. Clients experienced confusion due to the switch between measures each week, and missed sessions made it difficult for clinicians to track the required surveys each week.	With the reduction in items after replacing the peer and family scales, the 4 updated measures were consolidated into a single survey administered per session.	A single survey is simple and intuitive.
	3	Resource development	Clinicians required support resources after training: a reference explaining each scale in the measurement set, a written version of the technical and workflow components, a guide for documenting MBC^a^ in client charts, and an FAQ^b^ page.	The 4 resources mentioned were developed and consolidated into a single document, the “MBC Cheat Sheet,” and shared with clinicians.	Key support resources should be developed and shared following training.
	4	Ongoing training and consultation	Clinicians faced difficulties in three key areas postlaunch: discussing downward trends and discouraged clients, generating buy-in for resistant clients, and completing the survey with specific clients (younger, neurodivergent, etc).	A one-time follow-up training session was conducted by the external subject matter expert 4 months after launch. The training included targeted guidance, role plays, and question and answer.	Feedback–driven follow-up trainings are useful for postlaunch support.
**Study 2**					
	5	Survey language optimization	Clinicians reported some survey items did not include enough response options to capture a wide range of client experiences (eg, some clients do not take any medications or are not enrolled in school or working).	Survey language was adjusted in several places (ex. replaced “I worried about doing well in school” with “I worried about doing well in my responsibilities (eg, school or work).”	Item language and response options should be tailored to capture a wide range of client experiences
	6	Graph features optimization	The data display line graphs were difficult to follow- lines for individual items often overlapped, and colors were not distinct. The guiding language on each graph (eg, higher score is better) carried connotations linked to the words “better” or “worse.”	The displays were changed from line graphs to stacked bar graphs, and colors were adjusted for distinctiveness. Guiding language was changed from “higher score is better” to “higher score indicates symptom improvement.”	Data displays should be easy to follow, and language shared with clients should be neutral in tone.
	7	Communication channel creation	Clinicians needed a streamlined way to quickly communicate suggested changes and flag technical issues to the task group. Clinicians needed a way to consult with their peers, for example, troubleshooting client cases, asking for tips, etc.	A communication channel, staffed by 2 task group members, was developed within our messaging platform, including all clinicians and the task group. Clinicians could also respond to peer inquiries when appropriate.	A centralized channel enables quick communication and issue resolution, and facilitates idea sharing.

^a^MBC: measurement-based care.

^b^FAQ: frequently asked questions.

#### Cohort Characteristics, Training, and Launch

A single clinical cohort was chosen, encompassing 25 clinicians led by 1 clinical director and 2 supervisors. This cohort, the largest of the clinical teams, was chosen to capture the largest client population possible.

EBPAS-15 data were once again collected to assess clinician attitudes going into study 2 and design the trainings accordingly. Average domain scores for this cohort were similar to scores from the first cohort of clinicians in study 1, indicating consistent attitudes toward EBP implementation in both groups (Table 1). Internal consistency (Cronbach α) for the EBPAS-15 global scale was good (α=0.81) and subscales ranged from acceptable to excellent (required=0.97; appeal=0.79; and openness=0.85) with somewhat lower reliability estimates for divergence (α=0.44).

The second clinician cohort received 4 hours of live training from the external SME, with adjustments, including training on optimized measures and survey cadence, live consultation for common concerns regarding MBC, and provision of MBC companion materials immediately after training. Given that the updated training also included content from the refresher training conducted with study 1’s clinicians, no additional refresher trainings by the SME were necessary. In addition, 3 individuals were identified as “MBC champions”—key leaders and MBC enthusiasts—from the first study (CFIR *innovation recipients* construct). Champions described their experiences with MBC at the start of the training, including stories of their clients who greatly benefitted from using MBC during treatment, to facilitate positive peer perspectives and generate enthusiasm among the new clinician group. In support of key learning 4 (Table 2), the desire for ongoing MBC consultation, MBC champions were also available to the new clinician group for any ongoing consultation needs. The new clinicians were able to access support from the MBC champions as needed throughout the duration of study 2, generally by contacting them directly for peer-to-peer support.

#### Data Analysis

The same metrics and data analysis methods as those used in study 1 were used in study 2 to determine implementation success and outcomes, including survey completion rate, the monthly clinician feedback survey, and client outcomes.

## Results

### Study 1

#### Key Learnings and Changes

Four major learnings emerged from study 1, with relevant changes implemented in the months following the launch. Notably, we learned the following: (1) the measurement set needed further refinements, (2) the survey cadence needed simplification, (3) clinicians required support resources, and (4) clinicians required ongoing training and case consultation. All key learnings (detailed in Table 2) came from the live feedback session discussions and qualitative questions on the monthly feedback survey.

#### Clinician Data

The average survey completion rate was 80.08% (3216/4016) in the 4 months following the launch—in other words, an MBC survey was completed in around 80% of therapy sessions. From the monthly feedback survey, the average score for each quantitative item (shown in Table 3) indicated neutral attitudes toward MBC, with average responses in the “neither agree nor disagree” category. These scores were recorded approximately 1 month after the launch. Qualitative data from the survey were promising, with sample clinician quotes organized by theme in Textbox 1.

**Table 3:** Average item scores on the clinician feedback survey recorded approximately 1 month after launch, for both study 1 (n=22) and study 2 (n=22).

Scale item	MBC^a^ study 1 score, mean	MBC study 2 score, mean
I like using MBC.	3.73	4
I am excited to use MBC.	3.55	3.9
MBC adds value to my clinical practice.	3.82	4.4
MBC seems relevant to my clients.	3.95	4.3
MBC fits well with my therapeutic approach.	3.63	4.1
MBC seems easy to use.	3.77	4.3
I think MBC is going to work well here at Charlie Health.	3.91	4.3

^a^MBC: measurement-based care.

**Qualitative data: sample clinician quotes regarding measurement-based care (MBC) successes and struggles, with insights from both study 1 and study 2.**

**Positive theme**

- Helpful tool for clinicians
  “It’s so thorough and provides wonderful direction for treatment goals.”
  “I like seeing the hard data around how things are going. It has opened up conversation about things I hadn’t known previously.”
- Prompts conversation and collaboration
  “I like being able to discuss with clients specific areas where they have or could show improvement.”
  “If a client is not willing to provide an update about symptoms in the past week, showing MBC helps start that conversation.”
- Encouraging for clients and families
  “For my clients who like having evidence, proof, or visual cues regarding progress, this has been a major source of hope.”
  “I like when clients have not realized their progress, but when they see the numbers, they realize ‘I am better, I do feel better’.”
- Empowering for clients
  “Gives the clients the power to make their choices and express themselves.”
  “Really puts the responsibility of change into the client’s hands.”

**Negative theme**

- Survey is difficult for some clients and situations
  “For clients with reading deficiencies, this assessment takes up 10 minutes of a valuable session.”
  “I struggle with bridging the gap for clients who experience alexithymia or similar struggles when answering relevant questions, largely with neurodivergent clients.”
  “When a client is in extreme distress or needing to process intense trauma experiences, it’s been hard to pull focus to MBC.”
- Comfort with incorporating data
  “I struggle with adjusting my personal therapeutic style to include a more quantitative focus.”
  “It makes the session feel research based versus therapeutic.”

#### Client Data

The final client dataset consisted of 3723 clients who had all been discharged, with admission dates between February 13, 2023 (the launch date for study 1), and December 31, 2023. Out of 3723 clients, for the 2 comparison groups, 1576 (42.3%) clients belonged to the MBC group, while 2147 (57.3%) clients belonged to the TAU group. The ages of this sample ranged from 10 to 33 years, with an average age of 19.4 years. Around 52.99% (1973/3723) of clients identified as female, 24.98% (930/3723) identified as male, and 13.98% (520/3723) identified as a gender minority group.

In comparing routine discharge rates of MBC versus TAU clients, we found that MBC clients had a 22% higher likelihood of discharging due to successful treatment completion, a statistically significant finding (*P*<.001).

In addition, in all 3 outcome measures (PHQ-9, GAD-7, and WHO-5), we observed statistically significant differences in symptom reduction between MBC and TAU clients (detailed in Table 4). MBC clients experienced statistically significantly greater improvements in symptoms by discharge. However, though these results hold statistical significance, they do not appear to represent clinically significant symptom change, as both the MBC and TAU groups had average intake and discharge scores within the same clinical categories for each measure (eg, for the PHQ-9, both groups averaged “moderate” depression at intake and “mild” depression at discharge).

Clients with missing data for any outcome measure (PHQ-9, GAD-7, and WHO-5) were excluded from the respective measure’s analysis but included in analyses for other measures if their data were available.

**Table 4:** Summary of improvement in PHQ-9^a^, GAD-7^b^, and WHO-5^c^ scores for MBC^d^ versus TAU^e^ clients, from both study 1 (n=3723) and study 2 (n=405).

			MBC					TAU				*P* value			*t* test (*df*)
			Clients, n		Score, mean (SD)		Clients, n			Score, mean (SD)					
**Study 1**															
	PHQ-9 (depression)	532		–8.27 (7.09)		707			–6.56 (7.05)		<.001		–4.21 (1140)		
	GAD-7 (anxiety)	534		–6.66 (6.09)		705			–5.66 (5.86)		.003		–2.93 (1124)		
	WHO-5 (well-being)	540		+27.08 (25.16)		713			+20.92 (27.22)		<.001		–4.12 (1203)		
**Study 2**															
	PHQ-9	266		–8.08 (6.64)		74			–6.88, (5.89)		.07		–1.50 (129)		
	GAD-7	276		–6.84 (5.92)		67			–5.40 (5.38)		.03		–1.93 (108)		
	WHO-5	272		+28.82 (29.13)		70			+23.37 (23.94)		.05		–1.62 (127)		

^a^PHQ-9: 9-item Patient Health Questionnaire Modified for Adolescents.

^b^GAD-7: 7-item Generalized Anxiety Disorder.

^c^WHO-5: World Health Organization- Five Well-Being Index.

^d^MBC: measurement-based care.

^e^TAU: treatment as usual.

### Study 2

#### Key Learnings and Changes

Study 2 clinicians shared fewer overarching concerns about the implementation of MBC and instead provided feedback to support smaller adjustments. Three key learnings arose and are included in Table 2 (key learnings 5-7). Notably, (1) some language on the survey needed improvement, (2) specific aspects of the data display graphs needed optimization, and (3) a more streamlined communication channel for all-things MBC was needed.

#### Data From Clinicians

The average survey completion rate for study 2 was 86.01% (4218/4904) in the 3 months following launch. As for the quantitative items on the monthly feedback survey, average scores for study 2 clinicians improved in comparison to study 1 clinician scores, with average scores for almost all questions falling in the “agree” category (Table 3). Representative quotes from the qualitative data collected in study 2 are available in Textbox 1.

#### Client Data

The client outcomes analysis for study 2 was done with a much smaller sample size, given that fewer clients had been discharged since the launch of study 2, just 4.5 months before the analysis. The final dataset consisted of 405 clients who had all been discharged, with admission dates between September 25, 2023 (launch date of study 2), and February 1, 2024. Out of 405 clients, for the 2 comparison groups, 315 (77.8%) clients belonged to the MBC group, while 90 (22.2%) clients belonged to the same clinical team but did not participate in MBC (TAU group). The ages of this sample ranged from 10 to 34 years, with an average age of 17.5 years. Around 51.1% (207/405) of the clients identified as female, 20% (81/405) identified as male, and 15.1% (61/405) identified as a gender minority group.

In comparing routine discharge rates of MBC versus TAU clients, the MBC clients showed a 29% higher likelihood of discharging because of successful treatment completion than TAU clients, a statistically significant finding (*P*<.001).

Score improvements from intake to discharge for the PHQ-9, GAD-7, and WHO-5 (Table 4) were statistically significantly greater for MBC clients in the GAD-7 and WHO-5. The PHQ-9 score difference bordered on statistical significance. Similar to study 1, the results do not appear to represent clinically significant symptom change, as both the MBC and TAU groups had average intake and discharge scores within the same clinical categories for each measure.

## Discussion

### Study 1

Feedback collected in monthly meetings was crucial to informing necessary learnings and improvements to the MBC process. We learned that all scales in the measurement set must be sensitive to change, that a single weekly survey is far simpler and more intuitive than an alternating pattern with different measures, and that resources and ongoing trainings are essential for clinician support in the months following the launch. Using these key learnings, we incorporated a number of essential changes and applied the updates to study 2 (CFIR *adapting* construct).

### Study 2

Similar to study 1, our feedback-driven approach helped guide important changes in study 2. Most notably, we learned that survey language and item response options should be designed to cater to a wide array of client experiences, data displays should be easy to follow and as intuitive as possible, and that a centralized communication channel facilitates the sharing of ideas and rapid feedback delivery.

The implementation of these changes not only resulted in a decrease in the volume of clinician feedback but clinicians also reported experiencing a significantly streamlined version of the MBC process. Given the lack of further feedback and the overall efficiency of the process by the end of study 2, no further pilot studies were conducted. Following the conclusion of study 2, the task group began planning for the full clinical launch of the improved MBC process across all care teams (250 clinicians and 4000 clients), which took place in February 2024.

In terms of client outcomes, the sample size used in the analyses for study 2 (n=405) was notably smaller than the sample used in study 1 (n=3723), given that fewer clients had been discharged from study 2 at the time of the analysis. This may account for the nonsignificant and marginally significant results seen in study 2 as compared with study 1.

### Overview

#### Background

This study sought to add to the literature by documenting the design and deployment of an MBC QI initiative within a youth-serving, remote IOP. Recognizing the large size of our clinical workforce and the number of clients served, conducting pilot testing before any clinical implementation is crucial to gather learnings and evaluate success. Thus, we conducted 2 pilot studies with several clinical cohorts, aimed at understanding and adapting the MBC process to best fit the needs of our clinical population before a full-scale rollout. In both studies, several critical iterations were made to streamline and improve the process as much as possible. The increasingly positive clinician feedback ultimately led to our confidence in planning a full-scale clinical rollout following the conclusion of study 2.

Across both studies, results supported the feasibility and clinician acceptability of a rigorous MBC process in a youth-serving, remote-first intermediate care setting.

#### Survey Completion Rate

While ideally surveys would be completed in every session, achieving a 100% completion rate is often impractical due to the acuity of the client population, where crisis de-escalation may prevent survey completion. Although no universal benchmark exists for satisfactory MBC survey completion rates in IOP settings, the literature suggests that ≥80% indicates success [45]. Both study cohorts exceeded this benchmark, suggesting strong clinician buy-in and adherence to the “collect” portion of the model. The nearly 5% improvement in completion rate in study 2 possibly stems from refinements made during study 1.

#### Clinician Feedback

We designed the monthly feedback survey to track clinician attitudes toward MBC after the launch. Comparing week 4 of study 1 to week 4 of study 2, we saw higher scores in the second cohort, indicating more positive attitudes and perceptions at the same relative time point. This improvement likely also results from the iterative changes made during study 1, which helped to streamline the MBC process. Pilot clinicians also seemed to appreciate the task group’s responsiveness to their feedback, highlighting the value of an iterative approach and ongoing provider feedback in any clinical implementation.

#### Client Outcomes Data

The significant increase in routine discharge rate for the MBC groups in both studies (22% and 29%) was the most promising finding, suggesting that MBC is positively correlated with increased treatment completion. The PHQ-9, GAD-7, and WHO-5 outcomes were also promising, suggesting that MBC may be positively correlated with symptom reduction. However, despite the statistical significance of these results, clinical significance was not observed. Since this was a series of pilots, we were aiming for high-quality implementation and not necessarily outcome improvement, but the statistical significance observed in these pilots was promising and we hope to see clinical significance at full launch.

Overall, these results suggest that MBC may be more effective than usual practice. Our work aligns with previous literature, where meta-analyses have discussed that MBC leads to faster client improvement, greater symptom reduction, and lower dropout rates [17,24]. While we did not examine treatment dropout rates, we did see promising results regarding treatment completion rates and symptom reduction. A key distinction is that while previous studies largely focused on traditional in-person individual psychotherapy [46,47], our research was conducted within the context of a remote IOP, specifically geared toward youth.

### Key Factors

We believe a few key elements proved advantageous in the pilot studies, aligning with the aforementioned strategies and supported by the literature [30,31]. Similar to previous literature, we found that the use of a rigorous implementation plan [30,31], the consistent collection of stakeholder feedback [31], and the development of local champions [30,31] were all factors that supported success. However, another key factor in our work was the emphasis we placed on culture and enthusiasm building—before, during, and after the pilot studies. This factor was integral to clinician buy-in and, ultimately, the high survey completion rates we observed in each study.

### Strengths and Limitations

Several factors greatly facilitated the implementation of the pilot studies. First, clinical leadership’s full investment and company resource allocation ensured sustained momentum and prevented potential setbacks. Second, given that Charlie Health is a growing organization, decisions are made quickly and improvements to programming can be implemented within days, in contrast to hospital-based systems or larger companies that often face additional bureaucracy. Implementing a pilot in this kind of agile setting means feedback can be addressed with concrete changes and iterations relatively quickly. Third, since Charlie Health serves a sizable and diverse client population, outcomes are reflective of a broad range of demographic and clinical factors, allowing us to understand the impact of a program like MBC more comprehensively than if studied with a smaller or more homogenous sample. However, even larger-scale studies are needed to validate and build on our preliminary findings. Finally, before this implementation, our providers were already using a data-driven protocol involving the WHO-5, as mentioned earlier. Consequently, our clinicians had some familiarity with collecting and using data in their sessions, which may have aided their adoption of MBC in these pilot studies. This may not be the case in other BHCO settings.

The project was not without limitations. First, although we developed an internal technical solution for real-time data collection and display, the process proved more challenging than anticipated. At times, there were issues with slow or inconsistent data population into the graphs, which proved frustrating for clinicians and clients. To mitigate the impact of these issues in the future, our engineering team developed a more sustainable technical solution for the full clinical launch. Second, direct client feedback was hard to surface. Given the high acuity of our client population, we typically aim to minimize outreach to clients that is not directly linked to their care. We were also mindful not to create a perception among clients that they were mere test subjects or that their weekly survey data were being used for research purposes rather than integral to their treatment. In future studies, direct client feedback, for example, through quantitative work (eg, surveys) and qualitative data (eg, interviews), could be highly insightful regarding client experience and satisfaction with MBC. For example, qualitative interviews could explore whether patients feel that MBC enhances their collaboration with clinicians compared with other settings.

Third, while we measured the survey completion rate as a way to assess the fidelity of the “collect” portion of the MBC model, we did not collect data on the “act” and “share” portions of the model. Future studies should include systematic audits of data display use during sessions and reviews of clinical documentation to determine fidelity to the entire model. Fourth, given that the focus of this paper was on the implementation process rather than client outcomes, we did not conduct in-depth statistical analyses controlling for a variety of factors that may have impacted client outcomes. In addition, while the comparison groups (MBC vs TAU clients) were drawn from the same clinician teams, they may differ in ways that were not studied before. Future studies should examine and control for factors, such as sociodemographic data, variability in diagnoses, or cooccurring conditions to further understand differences between the MBC and TAU conditions.

### Considerations and Applications

Two key aspects of Charlie Health—the remote and youth-focused nature of the program—are important to consider when interpreting the implementation success. First, clients who receive care do so in a remote environment, which may have positively influenced the feasibility of our approach. Remote care’s accessibility and convenience likely boosted participation and engagement in MBC activities for both clients and clinicians. In addition, remote care heavily uses technology, which can streamline assessments, data collection, and progress tracking as compared with the administrative burden associated with paper-based data collection, manual data entry, and management.

Second, Charlie Health is a youth-oriented space, which similarly could have affected the implementation positively. For example, youth are often more comfortable with technology than adults, making them more likely to embrace digital tools (web-based survey and data display dashboard) during therapy sessions. In addition, youths often tend to be more flexible and adaptable to new approaches and interventions, so they may be more open to participating in MBC assessments in general. For those who are resistant to MBC, parental involvement in treatment can provide additional support and accountability, facilitating the implementation of MBC protocols.

Considering these specific aspects of Charlie Health’s programming, the implementation presented in this paper holds significant applicability to other youth-oriented, remote treatment settings. As the number of remote BHCOs grows, our implementation of MBC can serve as a guide for other remote service providers. However, the generalizability of this implementation is not limited to these treatment settings alone. The strategies and learnings presented, such as those in Table 2 and Textbox 2, can be used to inform MBC implementation across other teams in many different settings. For instance, the emphasis on selecting measures sensitive to change (key learning 1), or the benefits of including an SME in the entire process, holds relevance for any MBC system.

**Key positive factors that may have aided the implementation of measurement-based care (MBC) at Charlie Health.**

**Planning and organization**

- Task group formation, the use of a project plan, and guidance from a project manager ensured cohesive and streamlined progress
- Inclusion of a subject matter expert ensured planning was grounded in expertise
- Weekly meetings provided regular updates and issue brainstorming

**Feedback collection and response**

- Direct clinician feedback guided key improvements, enhancing buy-in and demonstrating a commitment to minimize MBC integration burden
- Multiple avenues for clinicians to deliver feedback (surveys, group discussions, supervisor check-ins, communication channel) proved effective

**Culture and enthusiasm building**

- Socializing MBC to teams before and during pilots (chief clinical officer introductions, MBC champion messages) fostered buy-in and enthusiasm
- Sharing periodic updates on positive client outcomes built clinician confidence by showcasing the tangible impact of using MBC

### Future Directions

This study examined MBC within the individual psychotherapy component of a group-based remote program. Ongoing research is needed to understand the efficacy of MBC within the group-based psychotherapy portion of an intermediate program, where relatively little research exists [27,48].

In addition, not all of our clients struggle with the same symptoms, meaning a “one size fits all” approach in the form of weekly assessments given to every client may not be as beneficial as tailored assessments. In keeping with the Joint Commission Standard [20], standardized measures were used in the current implementation. However, previous research documents that clinicians prefer idiographic or individualized measure approaches [49], and thus, future research is needed to examine the use of these individualized measures within this care context.

Furthermore, there were a number of metrics we did not study, such as numbers around treatment engagement and client satisfaction. It remains to be seen how those outcomes are associated with MBC. This study was also specifically focused on obtaining a uniform and high-quality implementation of MBC in place rather than understanding how MBC helps the organization meet other organizational goals [50]. Future research can include monitoring aggregate patient outcomes to support ongoing data-driven QI and program development, something we are investigating as part of our full clinical launch.

Finally, the potential for MBC to improve therapeutic alliance and potentially address health disparities by elevating patient voices is understudied [18]. Further research is required to illuminate the relationship between MBC and antioppressive practices in health care at large, especially in mental health care where internalized stigma of mental illness and comfort with mental health providers can differ between demographic groups.

The integration of MBC at Charlie Health responds, in part, to the demand for more literature on the implementation of large-scale clinical initiatives within youth services, showcasing a commitment to advancing the understanding and efficacy of mental health interventions for this age group. As BHCOs increasingly embrace the integration of EBPs into their care models, we underscore the significance and feasibility of implementing MBC—a powerful means, with careful planning and consideration, to potentially elevate client outcomes and provide concrete quantitative guidance to clinicians. Furthermore, as mental health care evolves, the insights shared in this paper can serve as a valuable resource, exemplifying the potential benefits and best practices associated with the thoughtful integration of MBC. Ultimately, our aspiration is for this work to contribute to the ongoing enhancement of mental health services and the well-being of individuals receiving care within the BHCO setting.

## Abbreviations

BHCO: behavioral health care organization
CFIR: Consolidated Framework for Implementation Research
EBP: evidence-based practice
EBPAS-15: 15-item Evidence-Based Practice Attitudes Scale
GAD-7: 7-item Generalized Anxiety Disorder
IOP: intensive outpatient program
MBC: measurement-based care
PHQ-9: 9-item Patient Health Questionnaire Modified for Adolescents
PROMIS: Patient-Reported Outcomes Measurement Information System
QI: quality improvement
SME: subject matter expert
TAU: treatment as usual
WHO-5: World Health Organization-Five Well-Being Index

## Data Availability

Owing to the quality improvement nature of this study, participants did not agree to their data being shared publicly, so supporting data are not available.
